# Supplementary material for: High-throughput 5′ UTR engineering for enhanced protein production in non-viral gene therapies
Source: Nat Commun. 2021 Jul 6;12:4138. doi: 10.1038/s41467-021-24436-7 (PMC8260622; doi:10.1038/s41467-021-24436-7)
Supplement: Supplementary file 1 — Supplementary Information [file 41467_2021_24436_MOESM1_ESM.pdf]

SUPPORTING INFORMATION

High-Throughput 5' UTR Engineering for Enhanced Protein Production in Non-Viral Gene Therapies

Jicong Cao[1, 2, 3, 4, 7] , Eva Maria Novoa[4, 5, 6, 7 #], Zhizhuo Zhang[4, 5, 6 ,7], William C.W. Chen[1, 2, 3], Dianbo Liu[1, 4, 5], Gigi C G Choi[1, 2, 3 *], Alan S L Wong[1, 2, 3 *], Claudia Wehrspaun[1, 2, 3], Manolis Kellis[4, 5, 6 †], Timothy K Lu[1, 2, 3 ,4, 6 †]

[1]*Synthetic Biology Group, Research Laboratory of Electronics, Massachusetts Institute of Technology, Cambridge, MA 02139, USA.*

[2]*Department of Biological Engineering, Massachusetts Institute of Technology, Cambridge, MA 02139, USA.*

[3]*Synthetic Biology Center, Massachusetts Institute of Technology, Cambridge, MA 02139, USA.*

[4]*Broad Institute of MIT and Harvard, Cambridge, MA 02142, USA.*

[5]*Computer Science and Artificial Intelligence Laboratory, Massachusetts Institute of Technology, Cambridge, MA 02139, USA.*

[6]*Department of Electrical Engineering and Computer Science, Massachusetts Institute of Technology, Cambridge, MA 02139, USA.*

[7]*The authors contributed equally to this work.*

[#]*Present address: Center for Genomic Regulation (CRG), 08003 Barcelona, Spain.*

[*]*Present address: School of Biomedical Sciences, University of Hong Kong, Hong Kong, China*

[†]*Corresponding authors: manoli@mit.edu, timlu@mit.edu*

CONTENTS

1. Supplementary Figures 1-14

2. Supplementary Tables 1-3

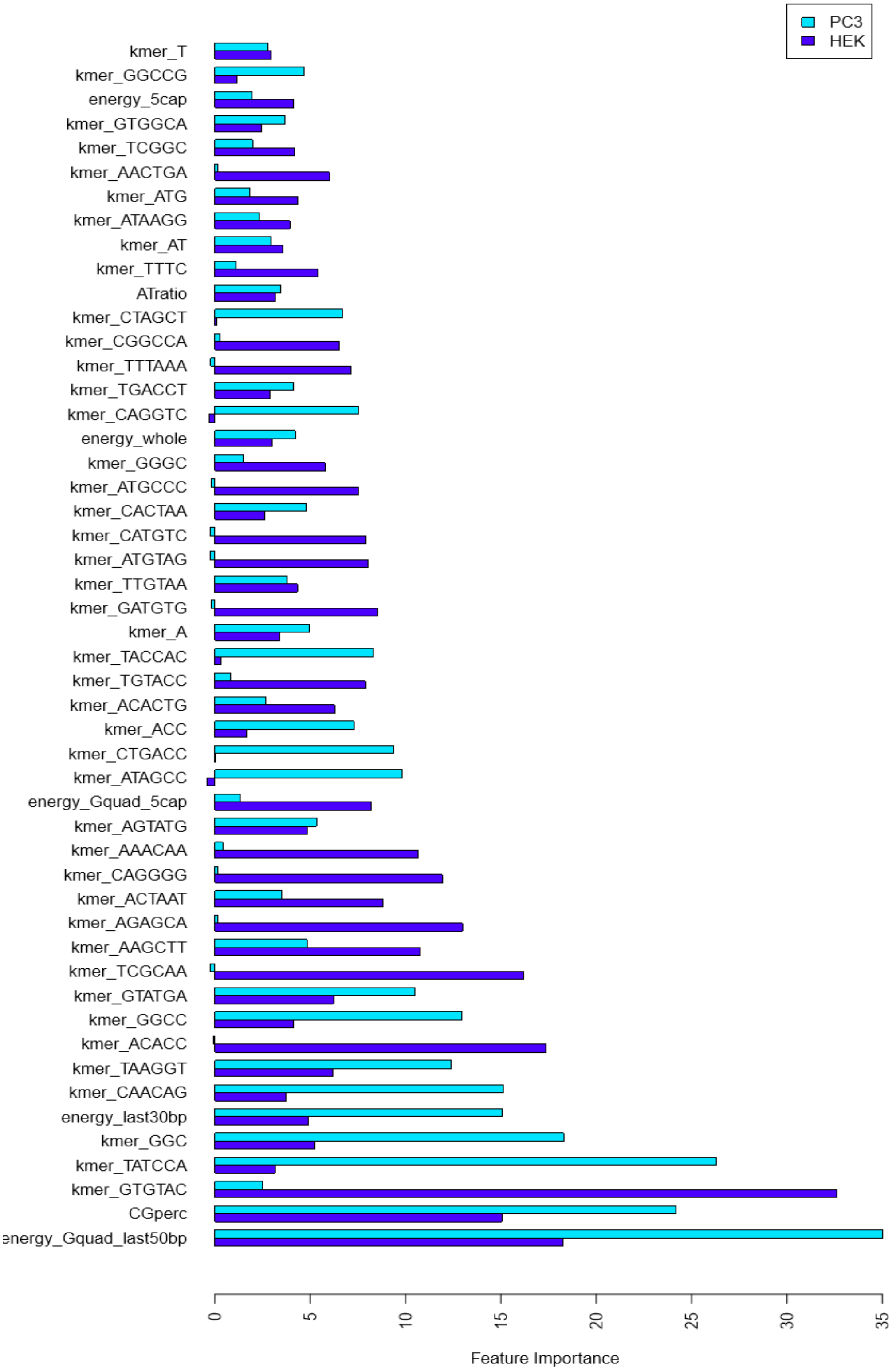

Supplementary Figure 1. Relative importance of the top-ranked individual features that were used for model training. The importance of features using as input PC3 Ribo-Seq and RNA-seq is shown in cyan, whereas those using as input HEK Ribo-Seq and RNA-seq are shown in blue.

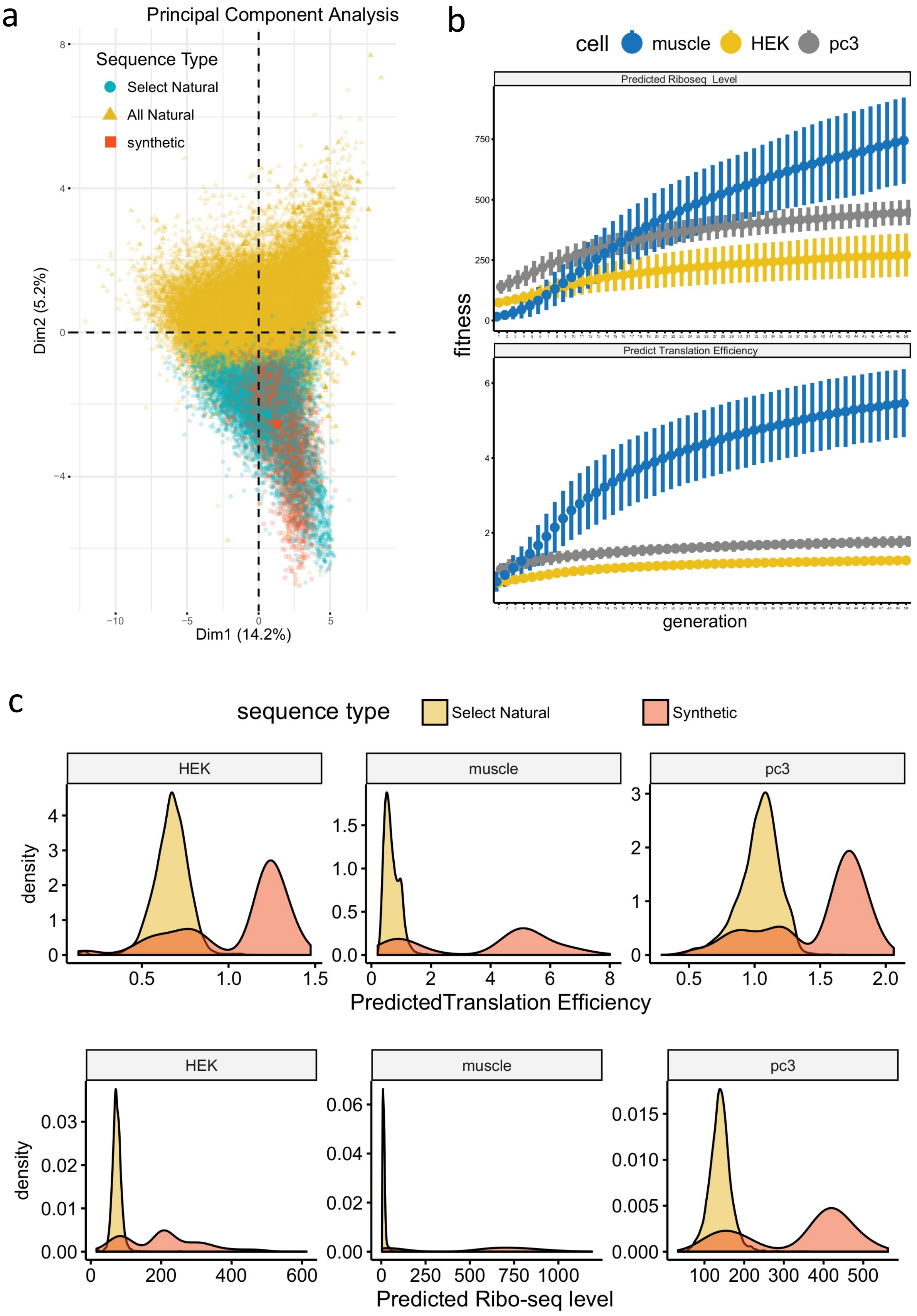
a
Principal Component Analysis
Sequence Type
Select Natural
All Natural
synthetic
Dim2 (5.2%)
Dim1 (14.2%)
b
cell
muscle
HEK
pc3
Predicted Riboseq Level
Predict Translation Efficiency
fitness
generation
c
sequence type
Select Natural
Synthetic
HEK
muscle
pc3
density
PredictedTranslation Efficiency
Predicted Ribo-seq level

Supplementary Figure 2. The generation and characterization of the synthetic 5' UTRs. a) Principal component (PC) analysis of different type of sequence designs. The PCs were derived from all extracted features from the union set of UTR sequences. The scatter plot shows the first PC on the x-axis and the second PC on the y-axis. All naturally occurring human UTR sequences (orange), selected 8415 naturally occurring UTR sequences (green), 3586 synthetic UTR sequences. b) The fitness change of each generation during the GA. Upper panel is based on the Ribo-seq level prediction model, and the 2nd panel is based on the translation efficiency prediction model. c) The upper row shows the predicted translational efficiency (TE) distribution of selected natural UTRs (yellow) and synthetic UTRs (red) across three tested cell types (HEK, Muscle, PC3). The lower row shows Ribo-seq level distribution of selected natural UTRs (yellow) and synthetic UTRs (red) across three tested cell types (HEK, Muscle, PC3).

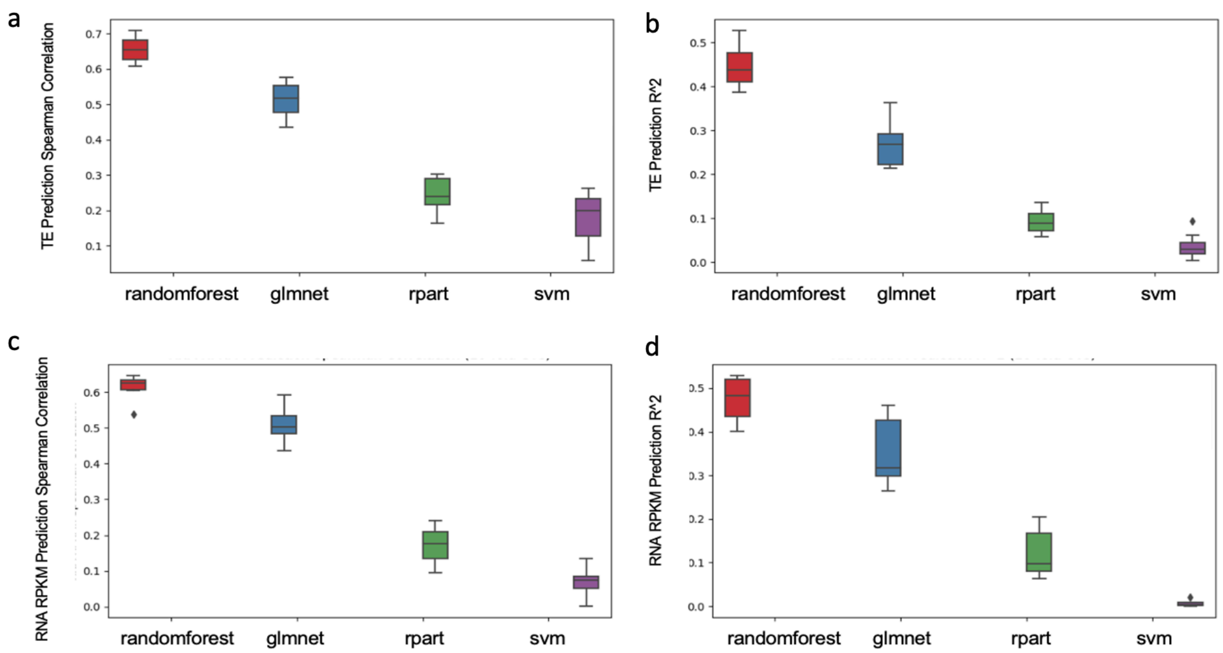

Supplementary Figure 3. The prediction comparisons between different models in HEK 293T cells. a) TE prediction spearman correlation; b) TE prediction R^2; c) RNA RPKM prediction spearman correlation; d) RNA RPKM prediction R^2.

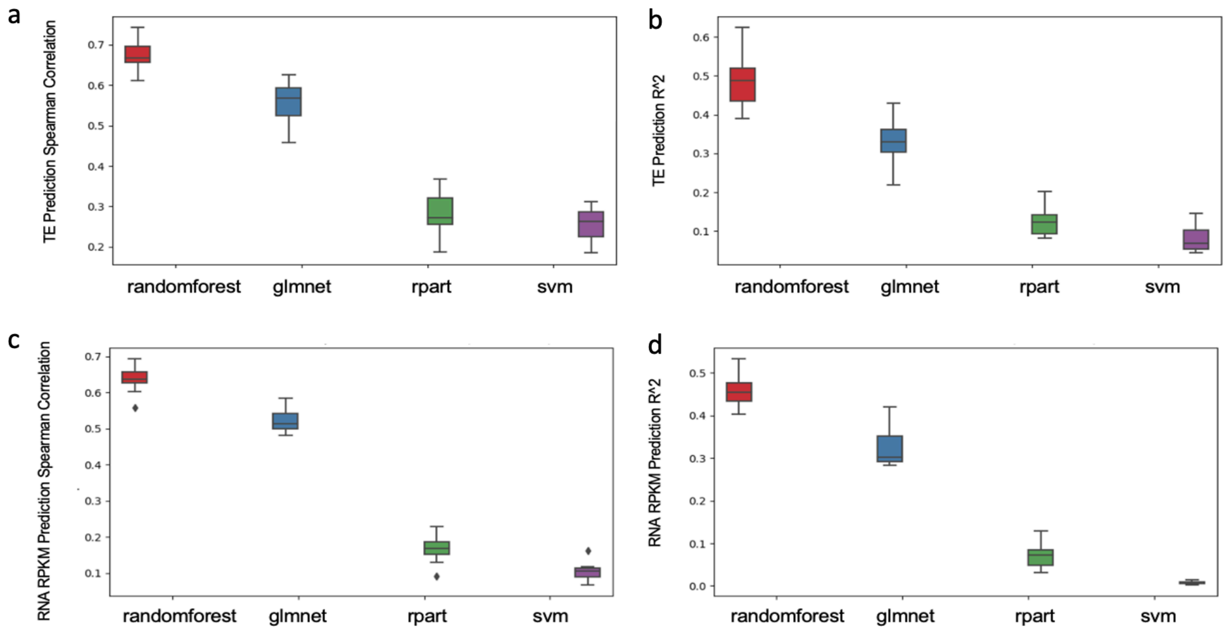

Supplementary Figure 4. The prediction comparisons between different models in PC3 cells. a) TE prediction spearman correlation; b) TE prediction R^2; c) RNA RPKM prediction spearman correlation; d) RNA RPKM prediction R^2.

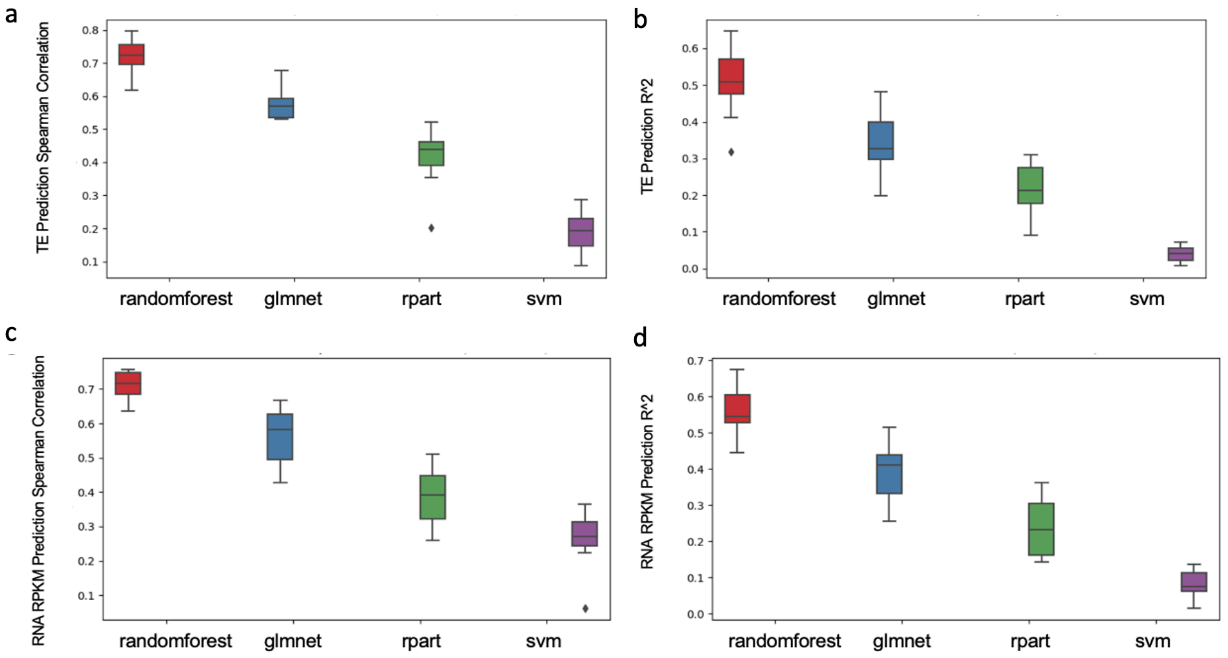

Supplementary Figure 5. The prediction comparisons between different models in muscle cells. a) TE prediction spearman correlation; b) TE prediction R^2; c) RNA RPKM prediction spearman correlation; d) RNA RPKM prediction R^2.

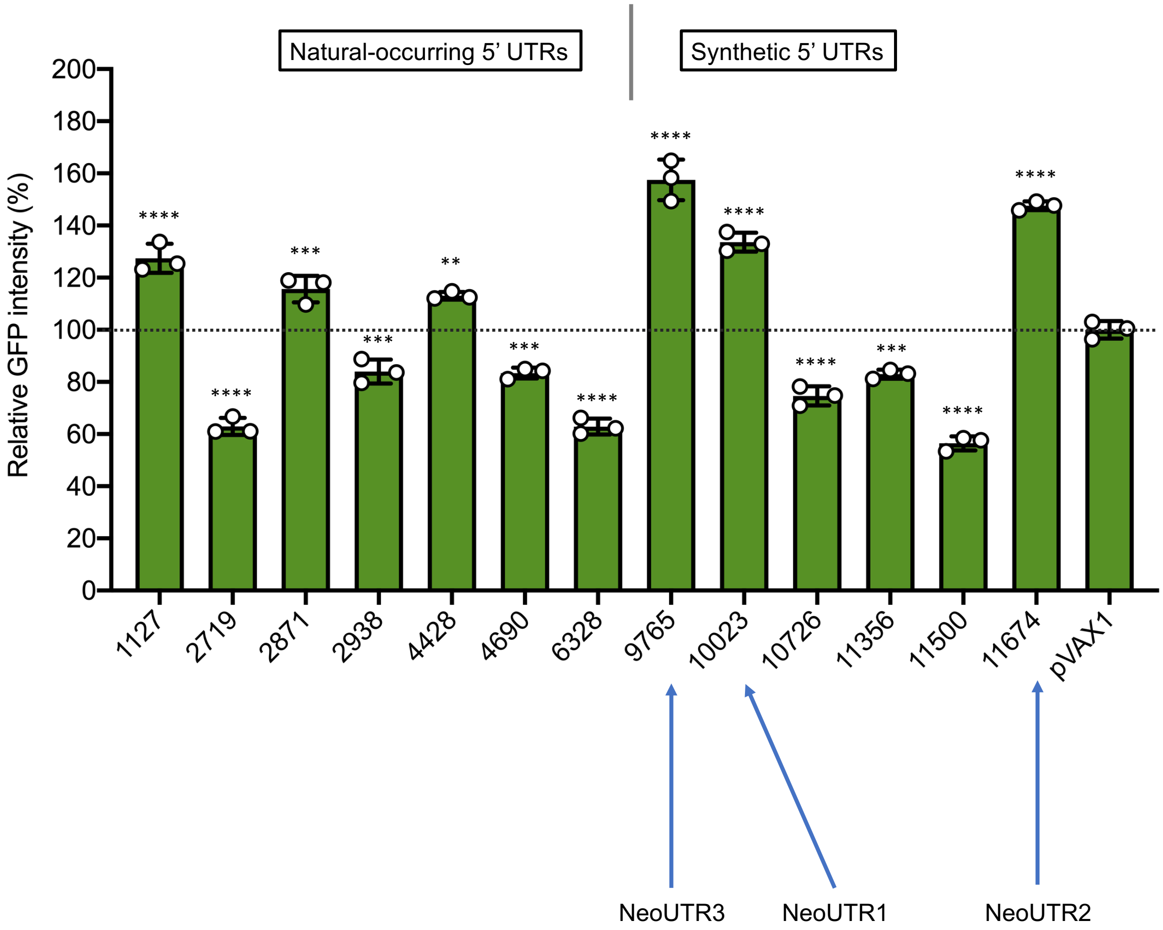

Supplementary Figure 6. The relative GFP intensities of the 13 candidate 5' UTRs in HEK 293T cells. Relative protein expression was normalized to that of the pVAX1-GFP plasmid, set as 100% and highlighted as a grey dotted line. Blue solid bars represent paired experimental groups. Source data are provided as a Source Data file. Statistical differences between groups were analyzed by ordinary one-way ANOVA with 95% confidence interval. Data are presented as mean values +/- SD for three biological replicates. The p-values for UTR1127, UTR2719, UTR2871, UTR2938, UTR4428, UTR4690, UTR6382, UTR9765, UTR10023, UTR10726, UTR11356, UTR11500, UTR11674 vs pVAX1 are <0.0001, <0.0001, 0.0005, 0.0003, 0.0036, 0.0002, <0.0001, <0.0001, <0.0001, <0.0001, 0.0002, <0.0001 and <0.0001. (*p<0.05; **p<0.01; ***p<0.001; ****p<0.0001)

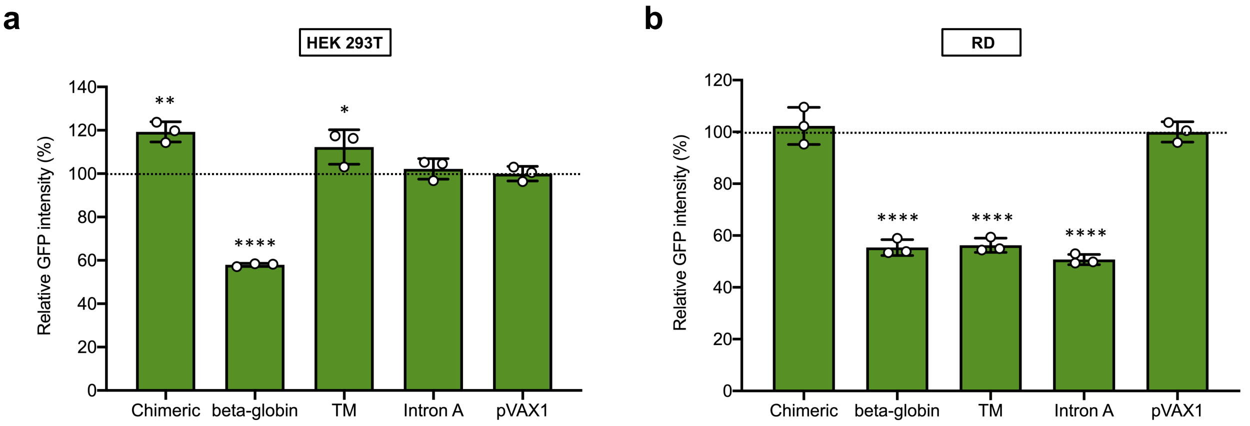

Supplementary Figure 7. The relative GFP intensities of the plasmids with different introns in HEK 293T (a) and RD (b) cells. Relative protein expression was normalized to that of the pVAX1-GFP plasmid, set as 100% and highlighted as a grey dotted line. Source data are provided as a Source Data file. Statistical differences between groups were analyzed by ordinary one-way ANOVA with 95% confidence interval. Data are presented as mean values +/- SD for three biological replicates. In HEK 293T cell lines, the p-values for Chimeric, beta-globin, TM and Intron A vs pVAX1 are 0.0023, <0.0001, 0.0368 and 0.9461. In RD cell lines, the p-values for Chimeric, beta-globin, TM and Intron A vs pVAX1 are 0.8948, <0.0001, <0.0001 and <0.0001. (*p<0.05; **p<0.01; ***p<0.001; ****p<0.0001 vs pVAX1)

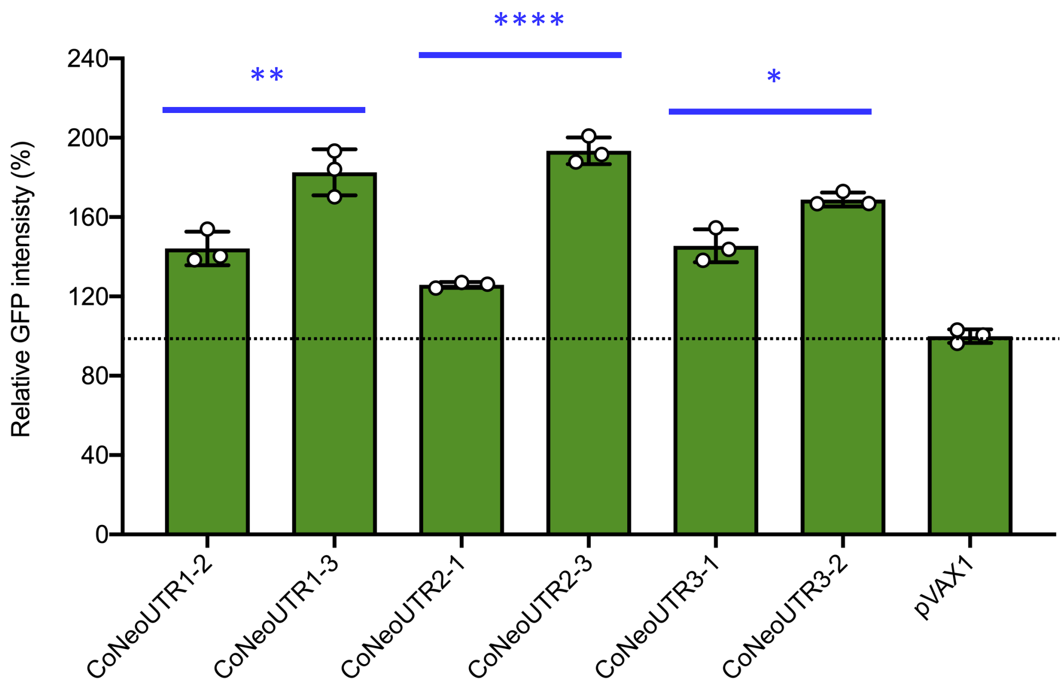

Supplementary Figure 8. The comparison of the fluorescence intensity attained with the combinatorial artificial UTRs. Relative protein expression was normalized to that of the pVAX1-GFP plasmid, set as 100% and highlighted as a grey dotted line. Source data are provided as a Source Data file. Statistical differences between groups were analyzed by unpaired t-test. Data are presented as mean values +/- SD for three biological replicates. The two-tailed p-value for CoNeoUTR1-2 vs CoNeoUTR1-3 is 0.0099. The two-tailed p-value for CoNeoUTR1-2 vs CoNeoUTR1-3 is <0.0001. The two-tailed p-value for CoNeoUTR1-2 vs CoNeoUTR1-3 is 0.0110. (*p<0.05; **p<0.01; ***p<0.001; ****p<0.0001)

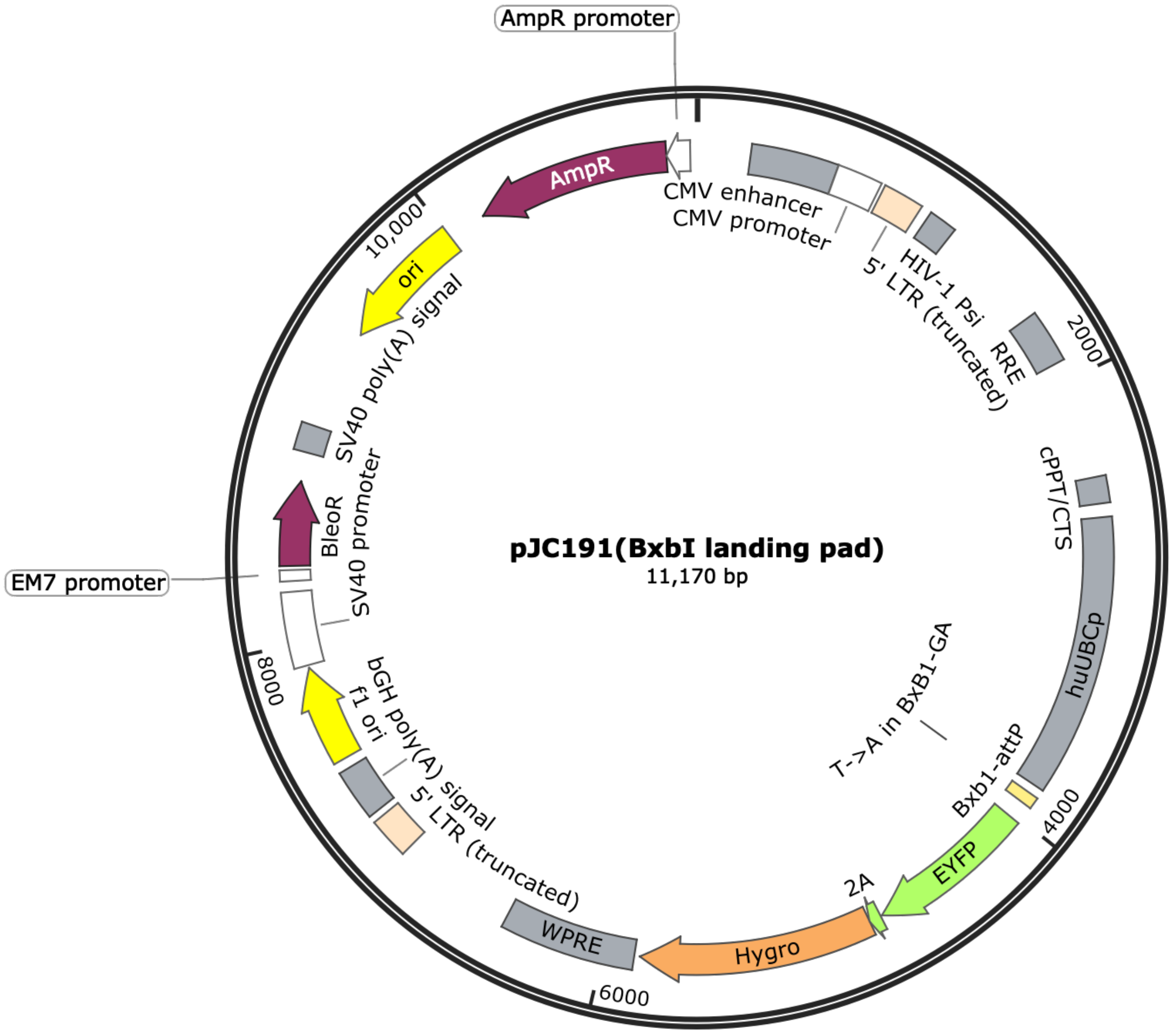

Supplementary Figure 9. Plasmid map of pJC191(BxbI landing pad)

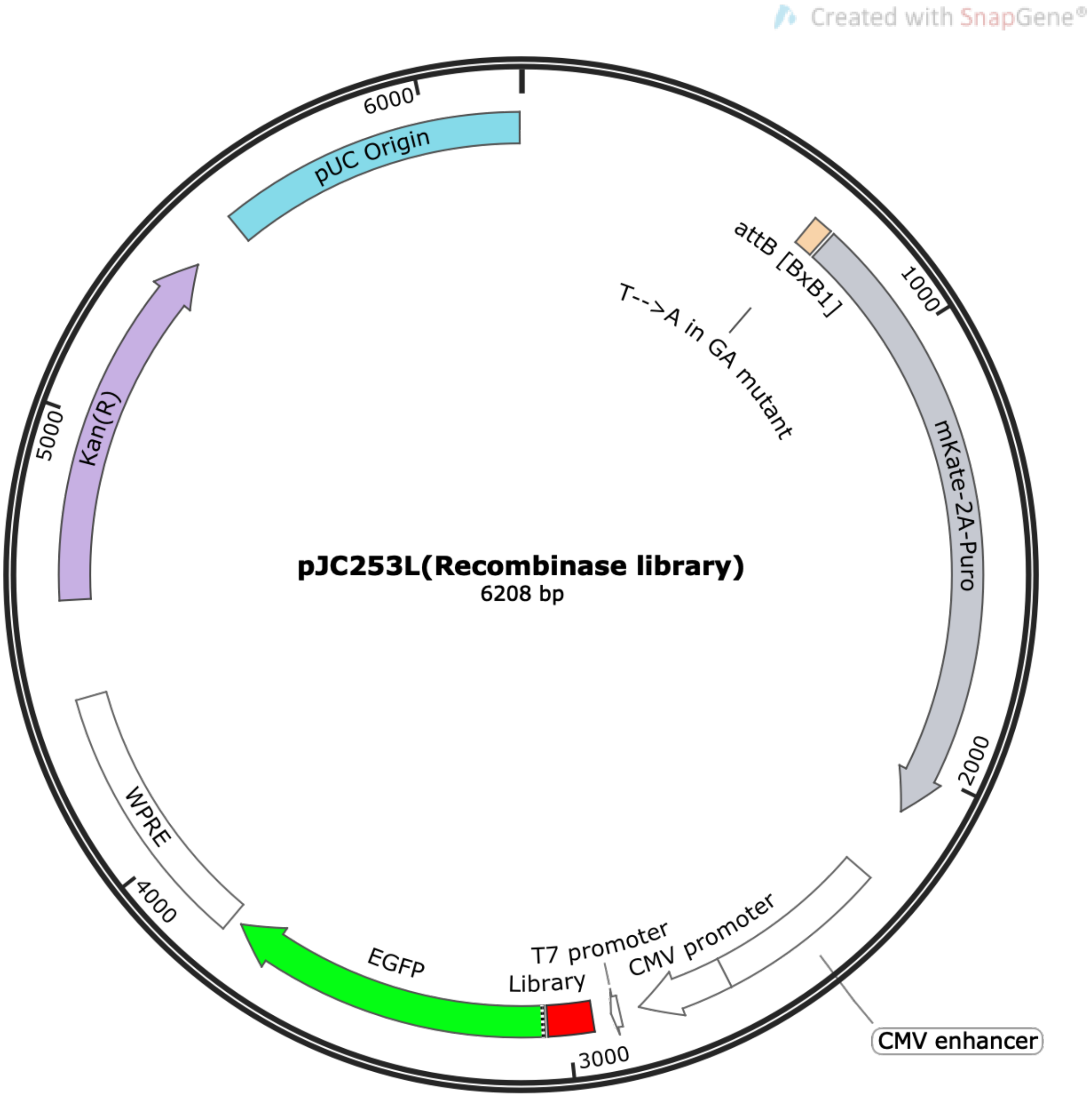

Supplementary Figure 10. Plasmid map of pJC253L (Recombinase library)

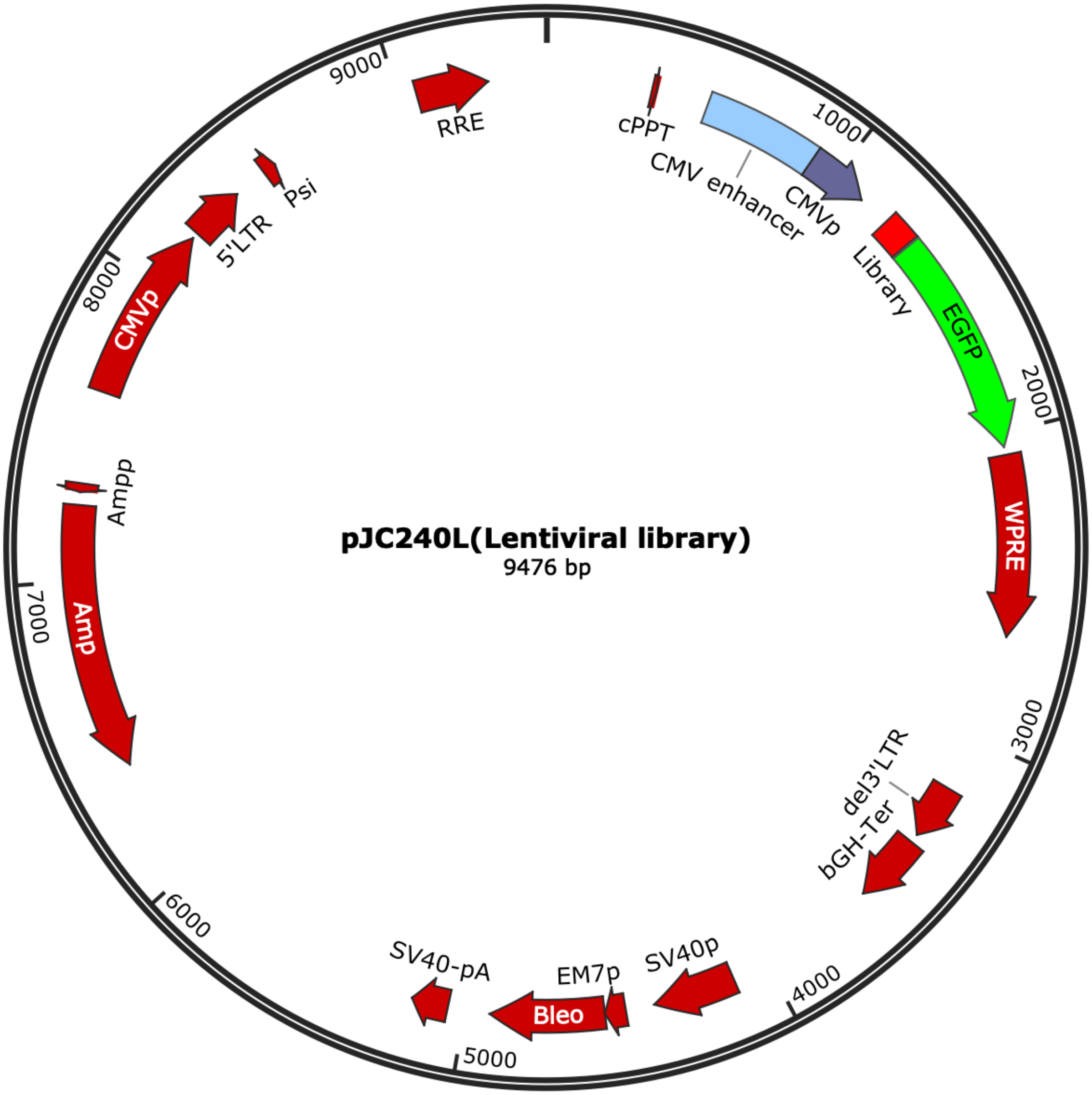

Supplementary Figure 11. Plasmid map of pJC240L (Lentiviral library)

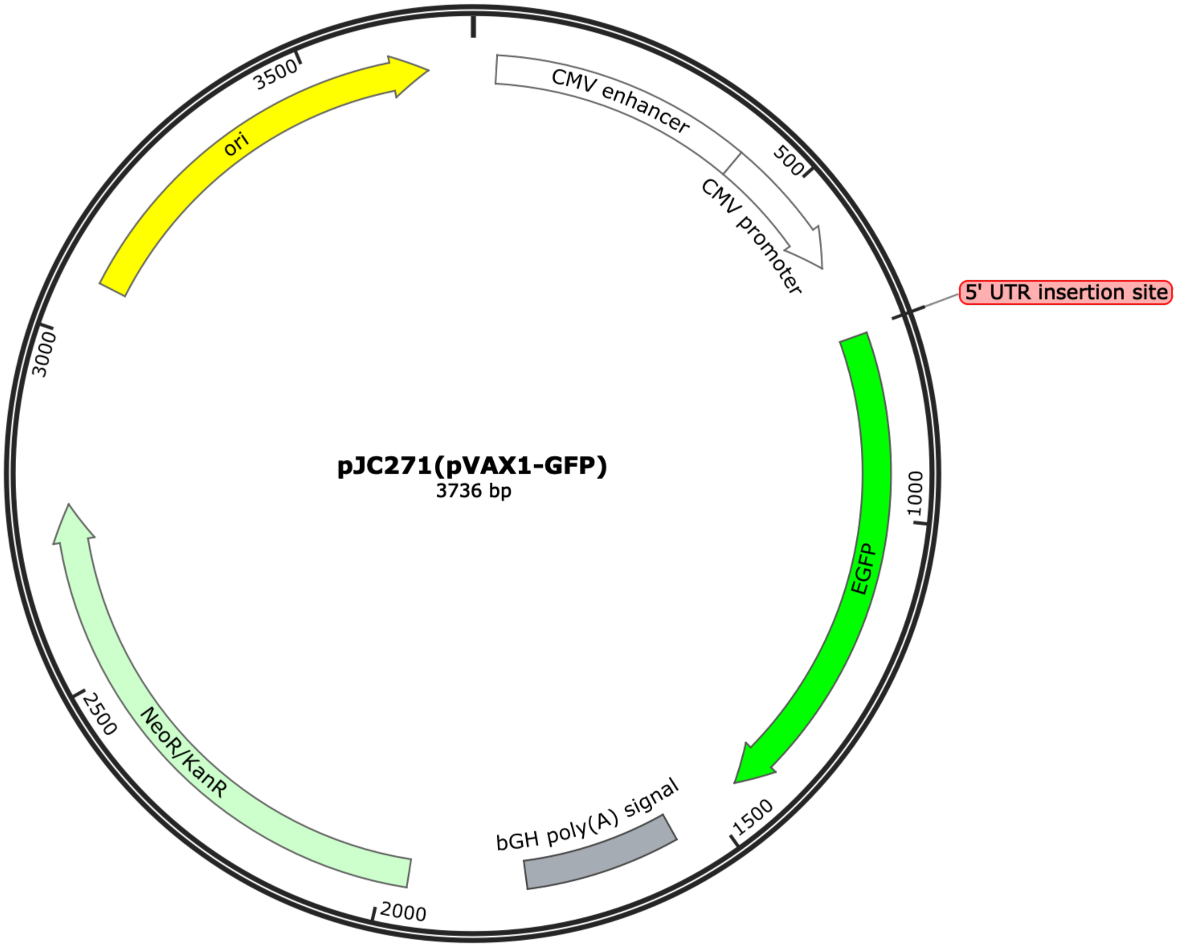

Supplementary Figure 12 Plasmid map of pJC271 (pVAX1-GFP)

Legend:

Human CMV promoter.

Leader sequence (the full-length 5’ UTR)

Transcription start site

5’ UTR candidates

Kozak sequence

GFP gene

GACATTGATTATTGACTAGTTATTAATAGTAATCAATTACGGGGTCATTAGTTCATAGCCCATATATGGAGT
TCCGCGTTACATAACTTACGGTAAATGGCCCGCCTGGCTGACCGCCCAACGACCCCCGCCCATTGACGTCAA
TAATGACGTATGTTCCCATAGTAACGCCAATAGGGACTTTCCATTGACGTCAATGGGTGGACTATTTACGGT
AAACTGCCCACTTGGCAGTACATCAAGTGTATCATATGCCAAGTACGCCCCCTATTGACGTCAATGACGGTA
AATGGCCCGCCTGGCATTATGCCCAGTACATGACCTTATGGGACTTTCCTACTTGGCAGTACATCTACGTAT
TAGTCATCGCTATTACCATGGTGATGCGGTTTTGGCAGTACATCAATGGGCGTGGATAGCGGTTTGACTCAC
GGGGATTTCCAAGTCTCCACCCCATTGACGTCAATGGGAGTTTGTTTTGGCACCAAAATCAACGGGACTTTC
CAAAATGTCGTAACAACTCCGCCCCATTGACGCAAATGGGCGGTAGGCGTGTACGGTGGGAGGTCTATATAA
GCAGAGCTCTCTGGCTAACTAGAGAACCCACTGCTTACTGGCTTATCGAAATTAATACGACTCACTATAGGG
AGACCCAAGCTGGCTAGCGTTTAAACTTAAGCTTGGTACCG---5’UTRcandidates---**GCCACCATG**G
TGAGCAAGGGCGAGGAGCTGTTCACCGGGGTGGTGCCCATCCTGGTCGAGCTGGACGGCGACGTAAACGGCC
ACAAGTTCAGCGTGTCCGGCGAGGGCGAGGGCGATGCCACCTACGGCAAGCTGACCCTGAAGTTCATCTGCA
CCACCGGCAAGCTGCCCGTGCCCTGGCCCACCCTCGTGACCACCCTGACCTACGGCGTGCAGTGCTTCAGCC
GCTACCCCGACCACATGAAGCAGCACGACTTCTTCAAGTCCGCCATGCCCGAAGGCTACGTCCAGGAGCGCA
CCATCTTCTTCAAGGACGACGGCAACTACAAGACCCGCGCCGAGGTGAAGTTCGAGGGCGACACCCTGGTGA
ACCGCATCGAGCTGAAGGGCATCGACTTCAAGGAGGACGGCAACATCCTGGGGCACAAGCTGGAGTACAACT
ACAACAGCCACAACGTCTATATCATGGCCGACAAGCAGAAGAACGGCATCAAGGTGAACTTCAAGATCCGCC
ACAACATCGAGGACGGCAGCGTGCAGCTCGCCGACCACTACCAGCAGAACACCCCCATCGGCGACGGCCCCG
TGCTGCTGCCCGACAACCACTACCTGAGCACCCAGTCCGCCCTGAGCAAAGACCCCAACGAGAAGCGCGATC
ACATGGTCCTGCTGGAGTTCGTGACCGCCGCCGGGATCACTCTCGGCATGGACGAGCTGTACAAG**TAA**

Supplementary Figure 13. Description of the features of pVAX1-5’ UTR-GFPs plasmids.

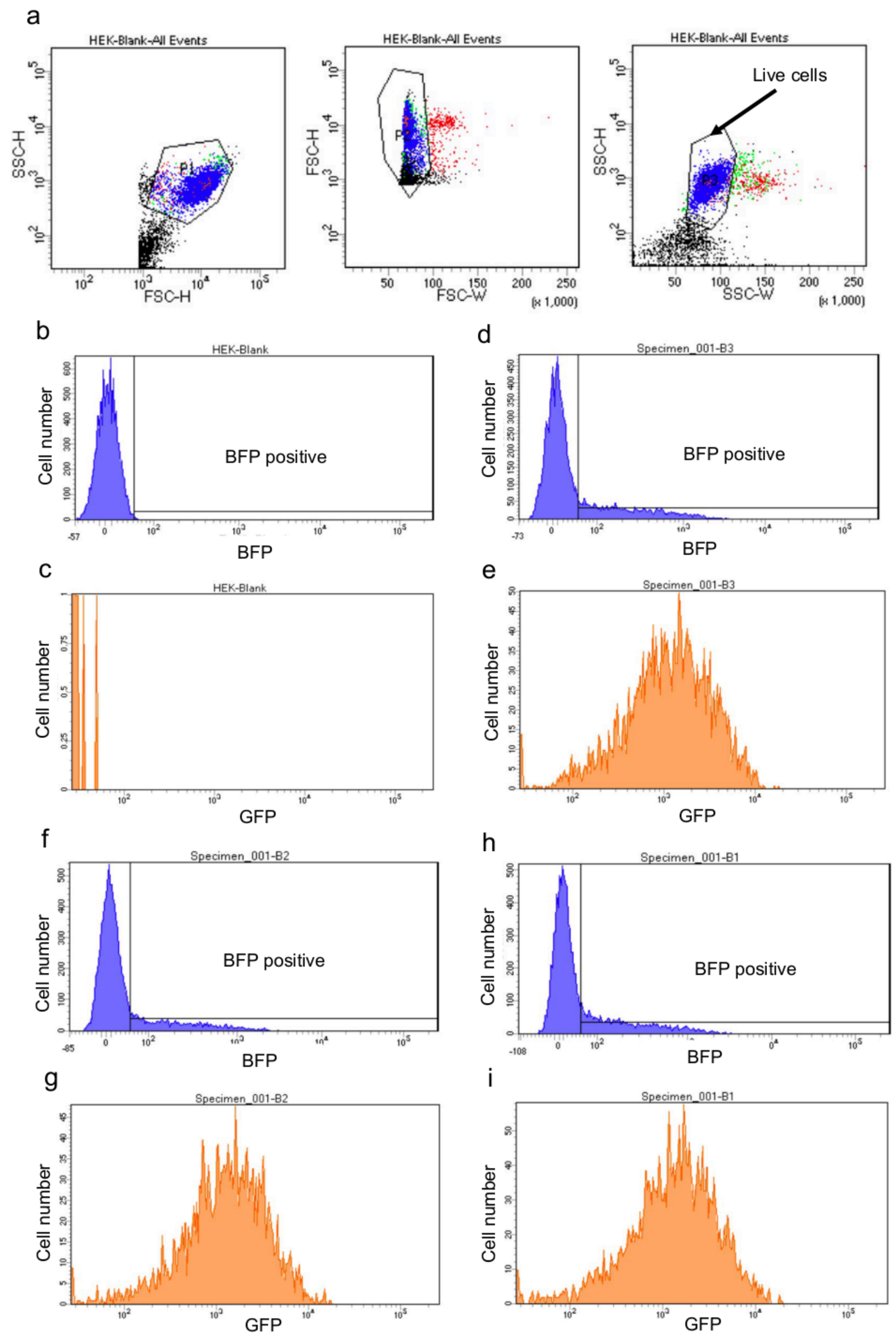
a
HEK-Blank-All Events
SSC-H
FSC-H
P1
FSC-W
(x 1,000)
P2
SSC-W
P3
Live cells
b
HEK-Blank
Cell number
BFP positive
BFP
c
GFP
d
Specimen_001-B3
e
f
Specimen_001-B2
g
h
Specimen_001-B1
i

Supplementary Figure 14. Flow cytometry analysis of protein expression for NeoUTR1, NeoUTR2, NeoUTR3 and pVAX1-GFP (Figure 4c). a) The gating strategy for live HEK 293T cells based on Forward Scatter (FSC) and Side Scatter (SSC). b,d,f,h) The histogram showing the selection of BFP positive cells transfected with BFP expression plasmid and pNeoUTR1-GFP (b), or pNeoUTR2-GFP (d), or pNeoUTR3-GFP (f), or pVAX1-GFP (h). c,e,g,i) The histogram showing GFP expression of BFP positive cells transfected with BFP expression plasmid and pNeoUTR1-GFP (c), or pNeoUTR2-GFP (e), or pNeoUTR3-GFP (g), or pVAX1-GFP (i).

Supplementary Table 1. The sequences of the primers used in this study.

Primer Names	Sequences
Library_F	TAAACTTAAGCTTGGTACCG
Library_R	CCCTTGCTCACCATGGTGGC
Backbone_F	ATGGTGAGCAAGGGCGAGGAGCTGTT
Backbone_R	AAGTCCCGTTGATTTTGGTGCCA
GFP_F	AAACTTAAGCTTGGTACCGGCCACCATGGTGAGCAAG
GFP_R	AGTGGATCCTAGAGTCGCGGCCGCTTTACTTGTACAGCTCGTCCATGCCGAGAGTGATCC
VEGF_F	AAACTTAAGCTTGGTACCGGCCACCATGGGAGTTAAGGTATTGTTCGCATTGATTTG
VEGF_R	AGTGGATCCTAGAGTCGCGGCCGCTTCACCGCCTCGGCTTGTC
CCL21_F	AAACTTAAGCTTGGTACCGGCCACCATGGCTCAATCCTTGGCGCTG
CCL21_R	AGTGGATCCTAGAGTCGCGGCCGCTTTATGGCCCTTTTGGCGTCTGG

Supplementary Table 2. The sequences of the thirteen 5’ UTR to validate. UTR 10023 was named after as NeoUTR1, UTR 11674 was named after as NeoUTR2, UTR 9765 was named after as NeoUTR3.

UTR IDs	Sequences
1127	GCATTCCAACCTTCCAGCCTGCGACCTGCGGAGAAAAAAAATTACTTATTTTCTTGCC CCATACATACCTTGAGGCGAGCAAAAAAATTAAATTTTAACC
2719	CCACGGCTACTGCGTCCACGTGGCGGTGGCGTGGGGACTCCCTGAAAGCAGAGCGGC AGGGCGCCCGGAAGTCGTGAGTCGAGTCTTCCCGGGCTAATCC
2871	GCCGGTGGCGGCAGGATACAGCGGCTTCTGCGCGACTTATAAGAGCTCCTTGTGCGGC GCCATTTTAAGCCTCTCGGTCTGTGGCAGCAGCGTTGGCCCG
2938	CCTGCTGAAGGGGCCCGACTGGATCCTGGGCGAGATCAAGACATCGGGTTTGAGGGG CCGTGGAGGCGCTGGCTTCCCCACTGGCCTCAAGTGGAGCTTC
4428	CTACAGAAACGAAAGAAAAAGTCTGTATAAGCCAAAGGTGTTCGGGAAGAAAATAAC CCCATTGCCTTGAGTTTGTAGGTGCCACTACTACTCTGGAAAA
4690	ACCGGAAAGAGGGTGGCTGAGGTGGGGGAGGAGCCCAAAAGGCATTGTGGGAGTAC AGCTCTTTCCTTTCCGTCTGGCGGCAGCCATCAGGTAAGCCAAG
6328	CTGCCCGACAAAATACATCAGAATTTCTCTTTAAGAACAATATCGGATCGATTAAAAA ATATATATATCGGATCAAATTGGGGGTACTTCAATACCTTGC
9765	CTTGTCTCGCTCCGGGGAACGCTCGGAAACTCCCGGCCGCCGCCACCCGCGTCTGTTC TGTTACACAAGGGAAGAAAAGCCGCTGCCGCACTCCGAGTGT
10023	CATTCTGTGGTCTGATCATCCTGTGGTTTCGTCGCCGCCATCCTCGTCGCGACACGCTG TTTTCGGTTCTCGGCCCGACGAGCCATCGCCATCCTACAGC
10726	ACCAACAACCAACAACAACATCCACACCAACAACAACGCTGAAAGTGGTGTTTGCTTT CTCCACCAGAAGGGCACACTTTCATCTAATTTGGGGTATCGC
11356	CACCAGCTCCTCCACTCTCACACCCAGGATTCACAACCCAGGAGTCTAGACCCCCAGC CCCTCCACACTCCCACCCAGGAACAACCCGGATAGGTCGGAC
11500	GCACCACACCCGCTGCAACCAGCCCCTAGACCACTCACACACTGCACAGGGACCAGC AACAACAACAAGACTCTCACAGAGAGTCAGCCGGCCTTCATAG
11674	CACTCGCGCTGCCATCACTCTTCCGCCGTCTTCGCCGCCATCCTCGGCGCGACTCGCTT CTTTCGGTTCTACCAGGTAGAGTCCGCCGCCATCCTCCACC

Supplementary Table 3. The sequences of the four introns used in this study.

**Intron Names**	**Sequences**
Chimeric	GTAAGTATCAAGGTTACAAGACAGGTTTAAGGAGGCCAATAGAAACTGGGCTTGTCGA<br>GACAGAGAAGATTCTTGCGTTTCTGATAGGCACCTATTGGTCTTACTGACATCCACTTTG<br>CCTTTCTCTCCACAG
Beta-globin	GTGAGTCTATGGGACCCTTGATGTTTTCTTTCCCCTTCTTTTCTATGGTTAAGTTCATGTC<br>ATAGGAAGGGGAGAAGTAACAGGGTACACATATTGACCAAATCAGGGTAATTTTGCAT<br>TTGTAATTTTAAAAAATGCTTTCTTCTTTTAATATACTTTTTTGTTTATCTTATTTCTAATA<br>CTTTCCCTAATCTCTTTCTTTCAGGGCAATAATGATACAATGTATCATGCCTCTTTGCACC<br>ATTCTAAAGAATAACAGTGATAATTTCTGGGTTAAGGCAATAGCAATATTTCTGCATAT<br>AAATATTTCTGCATATAAATTGTAACTGATGTAAGAGGTTTCATATTGCTAATAGCAGCT<br>ACAATCCAGCTACCATTCTGCTTTTATTTTATGGTTGGGATAAGGCTGGATTATTCTGAG<br>TCCAAGCTAGGCCCTTTTGCTAATCATGTTCATACCTCTTATCTTCCTCCCACAG
TM	CGTTTAGTGAACCGTCAGATCCTCACTCTCTTCCGCATCGCTGTCTGCGAGGGCCAGCTG<br>TTGGGCTCGCGGTTGAGGACAAACTCTTCGCGGTCTTTCCAGTACTCTTGGATCGGAAA<br>CCCGTCGGCCTCCGAACGGTACTCCGCCACCGAGGGACCTGAGCGAGTCCGCATCGACC<br>GGATCGGAAAACCTCTCGAGAAAGGCGTCTAACCAGTCACAGTCGCAAGGTAGGCTGA<br>GCACCGTGGCGGGCGGCAGCGGGTGGCGGTCGGGGTTGTTTCTGGCGGAGGTGCTGCTG<br>ATGATGTAATTAAAGTAGGCGGTCTTGAGACGGCGGATGGTCGAGGTGAGGTGTGGCA<br>GGCTTGAGATCCAGCTGTTGGGGTGAGTACTCCCTCTCAAAAGCGGGCATTACTTCTGC<br>GCTAAGATTGTCAGTTTCCAAAAACGAGGAGGATTTGATATTCACCTGGCCCGATCTGG<br>CCATACACTTGAGTGACAATGACATCCACTTTGCCTTTCTCTCCACAGGTGTCCACTCCC<br>AGGTCCAA
Intron A	GTAAGTACCGCCTATAGACTCTATAGGCACACCCCTTTGGCTCTTATGCATGCTATACTG<br>TTTTTGGCTTGGGGCCTATACACCCCCGCTTCCTTATGCTATAGGTGATGGTATAGCTTA<br>GCCTATAGGTGTGGGTTATTGACCATTATTGACCACTCCCCTATTGGTGACGATACTTTC<br>CATTACTAATCCATAACATGGCTCTTTGCCACAACTATCTCTATTGGCTATATGCCAATA<br>CTCTGTCCTTCAGAGACTGACACGGACTCTGTATTTTTACAGGATGGGGTCCCATTTATT<br>ATTTACAAATTCACATATACAACAACGCCGTCCCCCGTGCCCGCAGTTTTTATTAAACAT<br>AGCGTGGGATCTCCACGCGAATCTCGGGTACGTGTTCCGGACATGGGCTCTTCTCCGGT<br>AGCGGCGGAGCTTCCACATCCGAGCCCTGGTCCCATGCCTCCAGCGGCTCATGGTCGCT<br>CGGCAGCTCCTTGCTCCTAACAGTGGAGGCCAGACTTAGGCACAGCACAATGCCCACCA<br>CCACCAGTGTGCCGCACAAGGCCGTGGCGGTAGGGTATGTGTCTGAAAATGAGCGTGG<br>AGATTGGGCTCGCACGGCTGACGCAGATGGAAGACTTAAGGCAGCGGCAGAAGAAGAT<br>GCAGGCAGCTGAGTTGTTGTATTCTGATAAGAGTCAGAGGTAACTCCCGTTGCGGTGCT<br>GTTAACGGTGGAGGGCAGTGTAGTCTGAGCAGTACTCGTTGCTGCCGCGCGCGCCACCA<br>GACATAATAGCTGACAGACTAACAGACTGTTCCTTTCCATGGGTCTTTTCTGCAG
