# Supplementary material for: High-throughput 5′ UTR engineering for enhanced protein production in non-viral gene therapies
Source: Nat Commun. 2021 Jul 6;12:4138. doi: 10.1038/s41467-021-24436-7 (PMC8260622; doi:10.1038/s41467-021-24436-7)
Supplement: Supplementary file 2 — Reporting Summary [file 41467_2021_24436_MOESM2_ESM.pdf]

nature research

Corresponding author(s): Manolis Kellis, Timothy K Lu

Last updated by author(s): May 24, 2021

Reporting Summary

Nature Research wishes to improve the reproducibility of the work that we publish. This form provides structure for consistency and transparency in reporting. For further information on Nature Research policies, see Authors & Referees and the Editorial Policy Checklist .

Statistics

For all statistical analyses, confirm that the following items are present in the figure legend, table legend, main text, or Methods section.

n/a	Confirmed	
☐	☒	The exact sample size (*n*) for each experimental group/condition, given as a discrete number and unit of measurement
☐	☒	A statement on whether measurements were taken from distinct samples or whether the same sample was measured repeatedly
☐	☒	The statistical test(s) used AND whether they are one- or two-sided<br>*Only common tests should be described solely by name; describe more complex techniques in the Methods section.*
☒	☐	A description of all covariates tested
☒	☐	A description of any assumptions or corrections, such as tests of normality and adjustment for multiple comparisons
☐	☒	A full description of the statistical parameters including central tendency (e.g. means) or other basic estimates (e.g. regression coefficient) AND variation (e.g. standard deviation) or associated estimates of uncertainty (e.g. confidence intervals)
☐	☒	For null hypothesis testing, the test statistic (e.g. *F*, *t*, *r*) with confidence intervals, effect sizes, degrees of freedom and *P* value noted<br>*Give P values as exact values whenever suitable.*
☒	☐	For Bayesian analysis, information on the choice of priors and Markov chain Monte Carlo settings
☒	☐	For hierarchical and complex designs, identification of the appropriate level for tests and full reporting of outcomes
☐	☒	Estimates of effect sizes (e.g. Cohen's *d*, Pearson's *r*), indicating how they were calculated

Our web collection on statistics for biologists contains articles on many of the points above.

Software and code

Policy information about availability of computer code

Data collection	No software is used for data collection.
Data analysis	Statistical analyses were performed with GraphPad Prism 7.0.<br>BD Biosciences FACSDiva 8.0.1<br>Counts from next generation sequencing were analyzed using FastQC version 0.11.7 , FASTX-Toolkit version 0.0.13, Bowtie2 version 2.3.0, and SAMtools version 1.5. Counts were then processed by a custom R script and normalized using DESeq2 version 1.26.0.<br>The software versions used in the supplementary softwares are python 3.5, viennarna 2.1.9, R 3.5.1, biopython 1.72, r-randomforest 4.6.14, r-ga 3.2, r-seqinr 3.6.1, r-glmnet 2.0.16.

For manuscripts utilizing custom algorithms or software that are central to the research but not yet described in published literature, software must be made available to editors/reviewers. We strongly encourage code deposition in a community repository (e.g. GitHub). See the Nature Research guidelines for submitting code & software for further information.

Data

Policy information about availability of data

All manuscripts must include a data availability statement. This statement should provide the following information, where applicable:

- Accession codes, unique identifiers, or web links for publicly available datasets
- A list of figures that have associated raw data
- A description of any restrictions on data availability

Data supporting this study are presented in the main text and supplementary information, and are available from the corresponding authors upon request. The plasmid maps are available in FigShare (https://doi.org/10.6084/m9.figshare.14624472.v1). Raw FASTQ data has been deposited in the Gene Expression Omnibus, under the accession code GSE176581. Ribosome profiling data used in this work corresponds to GSE55195 (HEK293T), GSE35469 (PC3) and GSE56148 (muscle). This work used public RNAseq data from the GTEx database (https://gtexportal.org/). The processed data (extracted 5'UTR features) used for model training has been made publicly available in GitHub (https://github.com/zzz2010/5UTR_Optimizer).

Field-specific reporting

Please select the one below that is the best fit for your research. If you are not sure, read the appropriate sections before making your selection.

☒ Life sciences ☐ Behavioural & social sciences ☐ Ecological, evolutionary & environmental sciences

For a reference copy of the document with all sections, see nature.com/documents/nr-reporting-summary-flat.pdf

Life sciences study design

All studies must disclose on these points even when the disclosure is negative.

Sample size	Three biological replicates were performed for 5'UTR validation experiments using flow cytometry (N = 3). Four biological replicates were performed for ELISA experiments (N = 4). (e.g. Chavez, Nat. Methods, 2015).
Data exclusions	No data were excluded.
Replication	Data were analyzed over multiple biological replicates. The results were reproducible over at least two independent experiments.
Randomization	HEK 293T cells and their derivatives were used. As we performed experiments with a cell line with defined genetic background, there were no need for randomization.
Blinding	The investigators were not blinded. As we performed experiments with a cell line with defined genetic background, there were no need for blinding.

Reporting for specific materials, systems and methods

We require information from authors about some types of materials, experimental systems and methods used in many studies. Here, indicate whether each material, system or method listed is relevant to your study. If you are not sure if a list item applies to your research, read the appropriate section before selecting a response.

Materials & experimental systems

n/a	Involved in the study	
☒	☐	Antibodies
☐	☒	Eukaryotic cell lines
☒	☐	Palaeontology
☒	☐	Animals and other organisms
☒	☐	Human research participants
☒	☐	Clinical data

Methods

n/a	Involved in the study	
☒	☐	ChIP-seq
☐	☒	Flow cytometry
☒	☐	MRI-based neuroimaging

Eukaryotic cell lines

Policy information about cell lines

Cell line source(s)	HEK 293T cells, human rhabdomyosarcoma (RD) cells, human breast adenocarcinoma (MCF-7) Cells, and mouse C3H muscle myoblast (C2C12) cells were obtained from the American Type Culture Collection.
Authentication	The cell lines from ATCC were not authenticated because these cell lines were rigorously authenticated by ATCC. All cell lines were obtained from ATCC or collaborators and were expanded and frozen at low passages.
Mycoplasma contamination	Cell lines are not yet tested for mycoplasma contamination.
Commonly misidentified lines (See ICLAC register)	No commonly misidentified lines were used in this study.

Flow Cytometry

Plots

Confirm that:

- [x] The axis labels state the marker and fluorochrome used (e.g. CD4-FITC).
- [x] The axis scales are clearly visible. Include numbers along axes only for bottom left plot of group (a 'group' is an analysis of identical markers).
- [x] All plots are contour plots with outliers or pseudocolor plots.
- [x] A numerical value for number of cells or percentage (with statistics) is provided.

Methodology

Sample preparation	All cells were trypsinized or dissociated to single cells, filtered through 0.22um filter, and run through flow cytometer.
Instrument	BD Biosciences LSRII Fortessa cytometer
Software	BD Biosciences FACSDiva 8.0.1
Cell population abundance	All cell populations analyzed by the flow cytometer are highly abundant pure populations of cultured cell lines.
Gating strategy	Cells were first gated according to FSC-A and SSC-A parameters to identify the major viable population (generally > 90% of the cell population), followed by the measurement of the fluorescence intensity. An example is provided in Supplementary Fig. 14.

- [x] Tick this box to confirm that a figure exemplifying the gating strategy is provided in the Supplementary Information.
